# Recent Researches on Tetanus

**Published:** 1894-03

**Authors:** R. T. Hewlett

**Affiliations:** Demonstrator of Bacteriology in King’s College, London


### SELECTIONS.

### RECENT RESEARCHES ON TETANUS.

By R. T. Hewlett, M. D., Demonstrator of Bacteriology in King's College, London.

The record of the investigations into the nature of tetanus and the increase of our knowledge of that disease are remarkable illustrations of the progress of modern scientific research, of the advance from the unknown to the known. Ten, or even six, years ago the ethiology of tetanus was quite uncertain; the disease was regarded as a mysterious one, and many speculations were made as to its nature. No obvious or characteristic changes being met with after death, tetanus was regarded by many as a "functional" disease, a convenient term which commits us in no way and expresses little. Others believed that a primary lesion of the central nervous system might be the cause of the affection, while a few classed it with the "specific" diseases. Tetanus is now known to belong to the "acute infective diseases"—a causative agent in the form of a characteristic micro-organism has been isolated, the chemical products elaborated by this have been investigated, a great deal has been learnt as to the conditions necessary for its development in the body, and, lastly, a new method of treatment has been introduced.

Tetanus seems to have been known even in classical times, and it has long been recognized that lacerated or contused wounds, especially those soiled with dirt, are most liable to be followed by the disease, though slight and trivial injuries are not exempt, and cases occasionally occur (the so-called idiopathic ones) where no breach of continuity can be discovered.

This was the state of our knowledge until 1884, in which year Nicolaier produced tetanus in mice and rabbits by introducing garden earth beneath the skin; in the active earth, and also in the inoculation wounds, he found a peculiar micro-organism in the form of a bacillus. Nicolaier did not succeed in obtaining pure cultivations of this organism, but with his impure cultures he was able to produce tetanus in animals. In the same year Carle and Rattone showed that tetanus was an infective disease, that the bacillus of Nicolaier was present in the tissues and secretion of the wound in cases of traumatic tetanus in man, and that inoculation with the pus from such a wound produced tetanus in the lower animals. Rosenbach confirmed these observations in 1885.

Brieger, in 1886, obtained from the impure cultures of Nicolaier's bacillus chemical substances of the nature of alkaloids, two of which he named tetanine and tetano-toxine,—the former producing tetanic symptoms in mice, and the latter tremor, paralysis, and finally convulsions. It is interesting to note that Brieger has isolated tetanine from the amputated limb of a tetanic patient. A little later, Chantemesse and Widal by an elaborate method obtained the bacillus of Nicolaier in pure cultivation, but strange to say these pure cultures have lost the power of inducing tetanus, a fact which, with our present knowledge, can easily be explained. Kitasato, a Japanese observer working in Berlin, in 1889 successfully isolated the bacillus of Nicolaier in a simple and ingenious manner. This organism, the *Bacillus tetani* as it is now called, is met within the form of a fine rod, sometimes short, sometimes long, rounded at the ends, and motile. The short rods develop spores at one extremity; these are clear, shining, rounded bodies, four or five times the diameter of the rods from which they arise. The short rod with attached spore is very like a drum-stick and the organism has been termed the "drum stick bacillus." The *Bacillus tetani* is strictly anaërobic—that is, will not develop in the presence of oxygen, and has to be grown in an atmosphere of hydrogen; hence a good deal of the difficulty formerly experienced in obtaining pure cultivations. The *Bacillus tetani* forms spores at an earlier period than the other anaërobic species with which it is usually associated in wounds, &c. The spores of bacteria are more resistant than the fully developed organisms to heat, the latter being killed at a much lower temperature, and Kitasato availed himself of this fact in his method of isolation. He made cultures from the inoculation wound in a case of tetanus in a man, and so obtained impure cultivations just as Nicolaier had done. The cultures were examined microscopically at short intervals, and, as soon as the "drum-stick bacillus" had developed spores, were heated to 80 degrees C. for three-quarters of an hour. By this treatment all the fully developed organisms, including the tetanus *rods*, were killed, while the more resistant spores alone retained vitality, and inasmuch as the *Bacilius tetani* was the only organism which had formed spores, these remained alive and readily grew on being transferred to fresh nutrient media, pure cultivations being thus obtained. By continual cultivation, especially if the conditions are not rigidly anaërobic, the *Bacillus tetani* is apt to lose its virulence and fails to produce tetanus on inoculation, and this is probably the reason why the cultures of Chantemesse and Widal were inactive.

Some have supposed that the presence of other organisms is necessary for the *Bacillus tetani* to be active, and have suggested that these absorb free oxygen and so bring about an anaërobic condition. Though this does not seem to be the correct explanation, these adventitious organisms may play a part by diminishing the vitality of the tissues, and rendering them less able to cope with the *Bacillus tetani*.

The *Bacillus tetani*, unlike the *Bacillus anthracis* for example, seems to be localized to the wound or seat of inoculation, and is never found in the blood or tissues, internal organs or central nervous system. How, then, are the general symptoms of the disease to be accounted for? They are due to a "toxæmia" or blood-poisoning—the organism elaborates chemical poisons, which are spoken of as "toxines," locally at the site of inoculation, and these are absorbed into the system and produce the general effects. This fact explains how it is that the disease can sometimes be arrested by amputation or re-amputation, or by excision of a cicatrix. By these means the place where the toxines are being formed is removed, and, provided that the amount absorbed has not been too great, a cure is naturally effected. This treatment fails when a large and so fatal dose of the toxines has already been absorbed. The cure said to occasionally follow the division of the nerve or neıves near the wound is more difficult to explain. In some of these cases, doubtless, the favorable result may be an instance of "*post hoc*" and not of "*propter hoc*," for the disease, though generally, is not necessarily fatal. There is, however, another possible explanation. I have noticed that very small doses of the toxines appear to produce a marked local effect, the muscles in the region of the inoculation becoming powerfully contracted, while the general effects are inappreciable. So where neurotomy has been of service possibly the toxines may have travelled along the sheaths of the nerves for a short distance before entering the general circulation; on division of the nerve this path is blocked, the toxines no longer pass into the system, and the case being a light one, a favorable result ensues. The fact that the nerves in the neighborhood of the wound are often in a state of intense inflammation lends support to this suggestion.

The spores of the *Bacillus tetani* are widely distributed in the soil, though some districts seem to be free from them, hence the fact that tetanus does not appear to be met with in all localities.

Although the organism is so widely distributed, cases of the disease are comparatively rare. The explanation of this is that other conditions besides the presence of the bacillus are necessary. Thus, unless circumstances are favorable, the organism develops so slowly in a wound that the tissue cells obtain the mastery and remove the intruder before it has had time to multiply. Another factor also is the dose of the organism, for Watson Cheyne found that injection of fewer than 1,000 bacilli into rabbits did not cause death. So also if the tetanus *spores* be deprived of their adherent "toxines" and injected they do not seem to produce the disease, while if with these same spores a little lactic acid be injected, tetanic symptoms follow. This is the explanation of the loss of virulence so often met with in cultivations; the organism forms only small quantities of the toxines, and so little of the latter adheres to the bacilli that the tissue-cells are not weakened sufficiently to prevent them waging a successful contest.

In addition to alkaloidal bodies, the *Bacillus tetani* also forms an extremely poisonous substance of an albuminous nature, a "tox-albumin," and this seems to be the most important constituent of the "toxine" which gives rise to the manifestations of the disease. The tetanus toxine is readily destroyed by heating to 65 degrees C. for five minutes, by strong acids and alkalies, and by the action of light, direct sunlight completely destroying it and rendering it non-poisonous after fifteen to eighteen hours' exposure. In a cool dark place the toxine maintains its toxity almost indefinitely. Rabbits, guinea-pigs and mice, goats, sheep and horses are all susceptible to tetanus, the dog, fowl and pigeon are very refractory, and cattle are rarely attacked.

Two classes of cases of the disease are usually described—the traumatic, where there is an obvious wound, and the idiopathic, where apparently there is no breach of continuity. There is probably no real distinction between these; in the latter the channel of infection has been some trivial injury which has escaped observation. Some recent work on immunity with regard to tetanus is most interesting and of far-reaching importance, opening up a wide field in the direction of the treatment of infective diseases. It has been shown that by injecting gradually increasing doses of the toxines, provided the initial doses be sufficiently small, an animal finally becomes tolerant of enormous doses, and it acquires such a high degree of immunity, that tetanus can hardly be communicated to it. More marvellous still is the fact that the blood or blood-serum of such an immunised animal will, if injected, confer immunity on other animals, cure the disease when already in progress, and destroy the chemical poisons of tetanus if mixed with them. The method of immunisation is a comparatively simple one; the *Bacillus tetani* is grown in sterilized "bouillon," in an atmosphere of hydrogen at about 35 degrees C. for four or five weeks. The cultures are then filtered through porous porcelain; the filtrate is germ-free, but contains the chemical poisons elaborated by the organism and is extremely virulent.

The treatment is commenced by injecting subcutaneously increasing doses of a weakened virus, the above filtrate being mixed with a dilute solution of iodine in potassium iodide, whereby its virulence is much diminished; then increasing doses of the untreated filtrate are injected, and finally increasing doses of the untreated filtrate are administered intravenously. Animals after four or five injections become very refractory, but in order to prepare an active blood-serum—a blood-serum which is active in destroying the tetanus poison—the treatment must be prolonged. Rabbits, mice, sheep and horses can all be rendered refractory and yield an active blood-serum. The horse is the best animal, perhaps, to employ for the preparation of the "tetanus antitoxine"—the name given to this active blood-serum—but the treatment is tedious, lasting as long as two months, and necessitating twenty-five or thirty injections. When the blood is found to be sufficiently active, which is done by testing its activity on mice, the animal is bled, the blood allowed to coagulate, and the blood-serum collected and dried by evaporation *in vacuo*. In the dried state it retains its properties unaltered for long periods of time. This, then, is the method of preparing the now celebrated tetanus antitoxine. Behring and Kitasto, in 1890, found that the blood of a rabbit, rendered refractory to tetanus, was capable of destroying the toxines of tetanus when mixed with them, one or two drops of the blood-serum neutralising and rendering inert ten drops or so of the filtrate from a virulent culture after a contact of fifteen to twenty minutes' duration.

They also found that mice, which are very susceptible to tetanus, after inoculation with the blood-serum of a refractory animal, became refractory likewise; and, moreover, the onset of tetanus can be prevented if this blood-serum is injected soon after infection, and even a cure effected after symptoms have appeared. This method has now been applied to the treatment of several cases of tetanus in man; large doses of the antitoxine have been injected, and a cure has followed in some instances.

In an animal rendered artificially immune, it would seem that some substance is formed in the blood which has the property of neutralising the tetanus toxines. In a naturally immune animal, however, as a fowl, the blood has no such action, and when mixed with the tetanus toxines these are not neutralized, so that immunity in this case must be due to some other cause.

This brief sketch justifies, I think, my opening statement, and tetanus now ranks as one of the infective diseases about which we know most.

The antitoxine treatment appears to effect a cure in cases of tetanus, and as similar results have been obtained with regard to two other diseases—diphtheria and pneumonia—this "serum therapathy" seems to have a future before it.—*The Veterinarian.*

